# MicroRNAs and Periodontal Disease: Helpful Therapeutic Targets?

**DOI:** 10.34172/apb.2023.048

**Published:** 2022-07-02

**Authors:** Abdolhakim Palideh, Mostafa Vaghari-Tabari, Ali Nosrati Andevari, Durdi Qujeq, Zatollah Asemi, Forough Alemi, Hemmatollah Rouhani Otaghsara, Sona Rafieyan, Bahman Yousefi

**Affiliations:** ^1^Faculty of Dentistry, Babol University of Medical Sciences, Babol, Iran.; ^2^Department of Clinical Biochemistry and Laboratory Medicine, Faculty of Medicine, Tabriz University of Medical Sciences, Tabriz, Iran.; ^3^Immunology Research Center, Tabriz University of Medical Sciences, Tabriz, Iran.; ^4^Department of Biochemistry, Faculty of Medicine, Hormozgan University of Medical Sciences, Bandar Abbas, Iran.; ^5^Cellular and Molecular Biology Research Center (CMBRC), Health Research Institute, Babol University of Medical Sciences, Babol, Iran.; ^6^Department of Clinical Biochemistry, School of Medicine, Babol University of Medical Sciences, Babol, Iran.; ^7^Research Center for Biochemistry and Nutrition in Metabolic Diseases, Kashan University of Medical Sciences, Kashan, Iran.; ^8^Shahid Rajayee Hospital, Babol University of Medical Sciences, Babol, Iran.; ^9^Department of Oral and Maxillofacial Pathology, School of Dentistry, Zanjan University of Medical Sciences, Zanjan, Iran.

**Keywords:** miRNA, Periodontium, Gum, Gingival epithelial cells

## Abstract

Periodontal disease is the most common oral disease. This disease can be considered as an inflammatory disease. The immune response to bacteria accumulated in the gum line plays a key role in the pathogenesis of periodontal disease. In addition to immune cells, periodontal ligament cells and gingival epithelial cells are also involved in the pathogenesis of this disease. miRNAs which are small RNA molecules with around 22 nucleotides have a considerable relationship with the immune system affecting a wide range of immunological events. These small molecules are also in relation with periodontium tissues especially periodontal ligament cells. Extensive studies have been performed in recent years on the role of miRNAs in the pathogenesis of periodontal disease. In this review paper, we have reviewed the results of these studies and discussed the role of miRNAs in the immunopathogenesis of periodontal disease comprehensively. miRNAs play an important role in the pathogenesis of periodontal disease and maybe helpful therapeutic targets for the treatment of periodontal disease.

## Introduction

**Figure 1:**
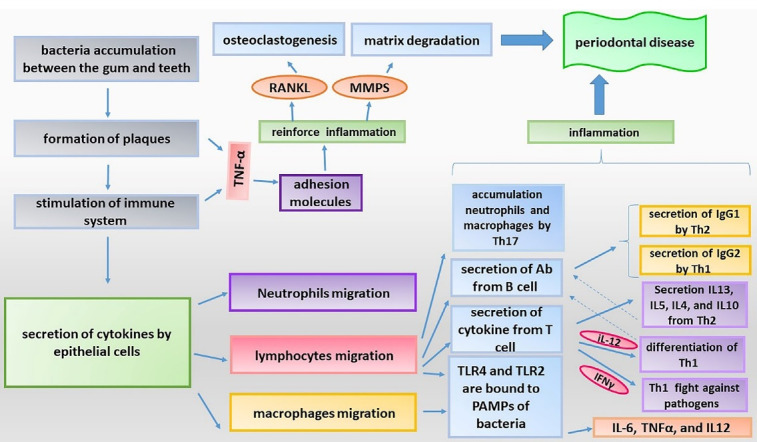
The periodontal disease progression mechanisms

 The inflammation process is useful process, which helps in removing pathogens and restoring and regenerating tissues. However, continuation of inflammatory reaction and development of frequent periods of inflammation bring about harmful effects. Indeed, an acute inflammatory response is a physiologic and useful event, and it is the chronic inflammation which is harmful and constitutes the basis of many human diseases.^1,2^ Periodontal disease is an inflammatory disease affecting the tissues around the teeth. This disease develops due to the reaction of the immune system to bacteria contaminating the tissues around the teeth.^3^ This disease is common whose prevalence in the public is around the teeth called periodontium become inflamed. Generally, gingiva, cementum (outer layer of dental root), and alveolar bone which maintain the teeth and periodontal ligaments which connect the teeth to the alveolar bone, are the constituents of the periodontium.^4^ The periodontal disease begins with gingivitis. Lack of proper mouth hygiene causes accumulation of bacteria in the gum line between the gum and teeth, causing formation of plaques (Figure 1). Accumulation of plaques causes stimulation of immune system, inflammatory reaction, and therefore sensitivity of the gum, which sometimes causes bleeding. If these plaques are not removed, they gradually become harder and spread below the gum line, and can cause constant stimulation of the immune system and development of chronic inflammation. Following these events, the gum tissue gradually retracts from the teeth and holes called periodontal pockets are developed, causing the bacteria to accumulate in these hollow spaces. In this way, the inflammation process is further reinforced, and continuation of this trend and constant production of inflammatory cytokines can cause osteoclastogenesis, loosening, and eventually detachment of the teeth.^5^ As mentioned earlier, the immune system and inflammatory process play a significant role in the development of this disease. In the primary stages of the disease, the origin of immune response is mostly the leukocytes residing in the periodontium, which physiologically try to remove the bacteria. Persistence of presence of bacteria causes secretion of cytokines by epithelial cells. Secretion of these cytokines causes stimulation of neutrophils recruitment.^6^ Periodontal disease has various risk factors including smoking, diabetes, having familial history, etc.^7^ The risk of developing this disease seems to increase with aging. Although the mechanism of development of periodontal disease is almost known, it is still unclear which bacteria are the direct cause of developing this disease. Nevertheless, some anaerobic gram-negative bacteria including *Porphyromonas gingivalis* have been propounded as the cause of this disease.^8,9^ In the rest of the paper, we deal with the importance of these bacteria and their LPS in the immunopathogenesis of periodontal disease. Development of the biology science in recent decades resulted in elucidation of the role of miRNAs in the process of inflammation. It was found that these small molecules play a significant role in the pathogenesis of a number of inflammation-associated disease.^10,11^ miRNAs are small non-coding RNA molecules with 20-22 nucleotides in length. These molecules were discovered in the early years of 1990s and gradually their different functions were recognized.^12^ The gene of miRNAs is coded by RNA polymerase II. The progenitor of miRNAs which has a loop-shaped structure is called pri-miRNA. This structure is influenced by an enzyme called Drosha and converts to pre-miRNA. Pre-miRNA then leaves the nucleus and after a series of interactions, it converts to mature miRNA. miRNAs by linking to the target mRNA cause stimulation of mRNA degradation or inhibits its translation.^11^ miRNAs play a significant role in the immunological interactions involved in the pathogenesis of periodontal disease, and are associated with different aspects of the immune function system ranging from differentiation of progenitor immune cells to production of cytokines and does signaling pathways involved in inflammatory response. In this review paper, attempts have been made to review the newest findings about the role of miRNAs in the immune response and pathogenesis of periodontal disease, and further investigate the role of these molecules into immunopathogenesis of this common disease.

## MiRNAs, periodontal cells, innate immunity and periodontal disease: A tight interconnection

 The innate immune response is the first defense line against pathogens. The innate immune system can detect different pathogens through pattern recognition receptors (PRRs) and respond to them. Indeed, detection of pathogens is the main step in initiating the innate immune response, which is very important in the pathogenesis of periodontal disease. PAMPs are identified by PRRs.^13,14^ Over the past few decades, several families of PRRs have been identified including Toll-like receptors (TLRs), nod-like receptors, and retinoic acid-inducible gene I (RIG-1)-like receptors.^15^ TLRs are the most prominent PRRs which are very important in periodontal disease. TLRs exist in a number of cells dependent on innate immune system including neutrophils, macrophages, dendritic cells, as well as some periodontium cells such as epithelial cells of the gum, periodontal ligament cells, and fibroblasts.^16-19^ TLR-2 and TLR-4 are the most well-known receptors in detecting the pattern in periodontal disease.^20^ The signaling pathways of TLRs, by activating NF-κB, cause production of pro-inflammatory cytokines and other pro-inflammatory factors such as cellular adhesion molecules, chemokines and prostaglandins.^21^ Although the innate immune response can protect an organism against pathogens, hyper response or a long term response as with what occurs in periodontal disease can be very detrimental. Therefore, the innate immune system should develop regulated and protective responses to different pathogen. With advances in the biomedical science and extensive studies performed to identify the precise mechanism of inflammatory diseases including periodontal disease, it was found that miRNAs have various roles in the regulation of immune response and inflammation and some miRNAs are involved in the progression of periodontitis.^22,23^ We further reviewed the newest findings about the role of miRNAs in innate immune response and periodontal disease. We also discussed about the role of these small molecules in adaptive immunity.

###  Gingival epithelial cells, immune system and miRNAs

 The gingival epithelial cells (GECs) are associated with periodontal health, such that, they act as physical barriers against microbial agents and play a significant role in innate immunity. These cells are constantly exposed to bacterial products, and in response to microbial agents, they secret pro-inflammatory mediators.^24^ HGECs can be involved in the regulation of periodontal inflammation through expressing miRNAs. One of the most important miRNAs in this regard is miR-203. In the introduction, we mentioned that some gram-negative bacteria including Porphyromonas gingivalis are suspect as a pathogen involved in developing periodontal disease, and this bacterium seems to be in relation with a number of miRNAs. One of these miRNAs is the miR-203. It seems that *P. gingivalis* increase the expression of miR-203 in HGECs, and this miRNA by inhibiting suppressor of cytokine signaling 3 (SOCS3) which is involved in compromising the innate immune response causes increased production of cytokines and progression of periodontitis.^25^ In the response to the Porphyromonas gingivalis stimulation, miR-584 seems to be also expressed by human gingival epithelial cells (HGECs), and through inhibiting the expression of lactoferrin receptor (LfR) in HGECs, attenuate the anti-inflammatory effects of human lactoferrin (hLf).^26^ Further, miR-155, miR-126, and miR-210 are also associated with periodontitis.^27^ Possibly, in response to microbial infections, miR-126, miR-155, and miR-210 are expressed in the GECs and regulate the inflammatory responses. Specifically, in these cells, miR-126 causes overexpression of IL-8, chemokine (C-C-C motif) Ligand 1 (CXCL1) chemokines, which are very crucial in recruiting immune cells especially neutrophils. However, miR-155 and miR-210 reduce the expression of IL-8 and CXCL1.^28^ Some studies have indicated that, the expression of miR-155 is enhanced by some bacterial pathogens such as LPS and bacterium derived nucleotides, pro-inflammatory cytokines, viral associated ligands such as synthetic TLR3 ligand poly (I:C), and CpG as well as IFN-B and IFN-γ, which are pro-inflammatory and antiviral cytokines.^29^ However, there are some contradictions as well. For example, some studies have indicated that in the gingival tissues of patients with periodontitis, in comparison to the gum tissues of healthy individuals, the expression of miR-155 decreases.^30^ Therefore, it seems that the roles of miR-155 are complex in the regulation of inflammatory processes. miR-155 act as an inhibitor of inflammation and inhibits expression of pro-inflammatory mediators; it can also be involved in enhancing the secretion of pro-inflammatory mediators and therefore development and progress of inflammation.

 Some cities have also revealed that miR-155 causes activation of the signaling pathway of TLR-4 and NF-κB, increased expression of IL8, and development and progress of inflammation through inhibiting the expression of SH2 domain containing inositol 5’ phosphate-1 (SHIP1) and signaling suppressor of cytokine-1 (SOCS-1) which are the molecules that inhibit the signaling pathway of TLR4.^31,32^ On the other hand, some studies have indicated that miR-155 attenuate TLRs signaling pathway by targeting the molecules associated with this pathway. miR-155 through reducing the expression of molecules such as TAK1 (Transforming growth factor beta-activated kinase 1)-binding protein 2 (TAB2), Myeloid differentiation primary response 88(mYD88), and IkB kinase (IKK) which are involved in reinforcing the TLRs signaling pathways, attenuate this signaling pathway and impair NF-κB activation.^33,34^ As stated earlier, pro-inflammatory cytokines stimulate inflammatory pathways and induce osteoclastogenesis.^35^ Therefore, these cytokines may be involved in the pathogenesis of periodontal disease. It seems that the overexpression of miR-155 which resulting from IFN-β, through targeting SOCS-1 and microphthalmia-associated transcription factor (MITF), which are two main regulators of osteoclastogenesis, inhibit differentiation of osteoclasts.^36^ Therefore, it seems that miR-155 is one of the miRNAs, which role in the immune interactions involved in periodontal disease and should be studied further. miR-142 is other miRNA associated with periodontal disease. It seems that in the response to the inflammation which caused by TNF-α, the expression of miR-142 significantly increases in HGECs.^37^ It seems that, the expression of miR-17 is in association with diminished expression of IL-8.^38^ IL-8 is an important chemokine involved in the recruitment of immune cells, so it is useful to study the association between miR-17 expression in HGECs and IL-8 secretion in periodontal disease. In overall, further studies can clarify more dimensions of the role of above mentioned miRNAs in the pathogenesis of periodontal disease.

###  Periodontal fibroblast, immune system and miRNAs 

**Figure 2:**
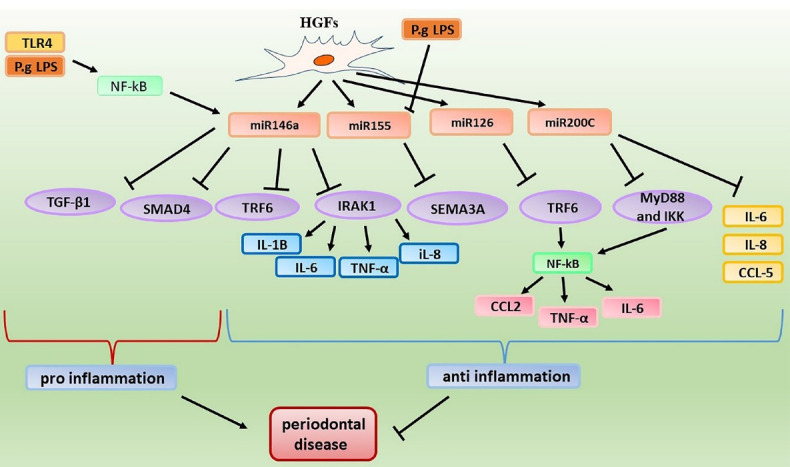
pro‐ and anti‐inflammatory effects of miRNAs in periodontal fibroblast cells

 Fibroblasts are connective tissue cells present in oral tissues such as gingival, periodontal ligament, and dental pulp tissues (Figure 2). In addition to their roles in integration and regeneration of periodontal tissue, fibroblasts may function as secondary immune cells, where they express the receptors and molecules associated with the innate immunity, and through identifying antigens, they cause production of inflammatory mediators.^39,40^ Different studies have been performed on the expression and effects of miR-146a and miR-155 in fibroblasts. It seems that the expression of miR-146a in human gingival fibroblasts (HGFs) stimulated by *P. gingivalis* LPS significantly increases in comparison to non-stimulated HGFs. miR-146a possibly through reducing the expression of IRAK1 repress the secretion of pro-inflammatory cytokines including IL-6, and TNF-α in HGFs.^41^ However, under pro-inflammatory conditions (stimulation with LPS) in gingival fibroblasts, the expression of miR-155 diminishes. It seems that miR-155 plays a significant role in the progression of periodontitis through inhibiting Semaphorin-3A (SEMA3A) which has an anti-inflammatory function.^22^ A study reported that miR-146a reduce the secretion of IL-8, IL-6, and IL-1B in *P. gingivialis* stimulated HPDL fibroblasts, through reducing the expression of TNF receptor associated factor 6 (TRAF6).^42^ In addition, it seems that this miRNA has also a considerable relationship with NF-κB, where activation of the TLR4 signaling pathway by Porphyromonas gingivialis LPS results in activation of NF-κB and increased expression of miRNA-146a in HGFs. This miRNA through inhibiting the activity of NF-κB may attenuate immune responses.^43^ As mentioned earlier, miR-146a can have useful effects in attenuating the periodontal disease progression. However, it also has some negative effects in this regard, which seem to be associated with its inhibitory effect on SMAD4 and TGF-B1 signaling pathway. TGF-β1 is involved in the restoration and regeneration of tissues. It has been shown that through stimulating the RNA synthesis as well as the proteins of periodontal ligament and gingival fibroblasts proteins, TGF-β1 contributes to regeneration of the periodontal tissue. SMAD4 plays an important role in the TGF-β1/SMAD signaling pathway.^44^

 Possibly, miR-146a through inhibiting the expression of SMAD4 attenuate the TGF-β1/SMAD signaling pathway and thus exert the inhibitory effect on fibroblasts differentiation.^45^ miR-126 is another miRNA which targets TRAF6. Concerning the effect of miR-126 on fibroblasts, a study suggested that miR-126 by reducing the expression of TRAF6, attenuate the NF-κB activity and thus repress the secretion of pro-inflammatory cytokines including IL-6, TNF-α, C-C Motif Chemokine Ligand 2 (CCL2) in HGFs.^46^ Other miRNAs expressed in fibroblasts are miR-223, miR-200, and miR-181a. It seems that pro-inflammatory cytokines including IL-1β, IL-6, and TNF-α increase the expression of miR-223 in HGFs. This miRNA through reducing the expression of IL-1β and IL-6 plays a protective role in the gingival fibroblasts and alleviate the inflammation.^47^ Concerning miR-200 some studies have also been performed. It seems that miR-200a can target C-X-C motif chemokine 12 (CXCL12). However, no significant difference in the expression of this miRNA was reported in oral fibroblasts following stimulation with stimuli.^48^ In addition, a study indicated that miR-200c through reducing the levels of MyD88 and IKK attenuate NF-κB activity.^49^

 It seems that miR-181a also directly targets IL-8.^50^ Therefore, miR-181a may attenuate progression of the mouth pulp inflammation, possibly through reducing IL8 expression; indeed, it may prevent progression of pulpitis to periodontitis.

###  Neutrophils, miRNAs and periodontal disease 

 Neutrophils are one of the main components of the innate immune system. These cells are the most abundant immune cells in the bloodstream. Neutrophils are activated under the influence of pathogens and are rapidly transferred to infection sites. Further, these cells have different antimicrobial functions, and are able to initiate inflammatory responses and produce chemokines to stimulate transmission of immune cells to the site of infection.^51^ miRNAs are in relation with these cells. Neutrophils can be involved in regulating the inflammatory responses in periodontal tissue through expressing the miRNAs. IL-8 importance regarding inflammation process was stated earlier. IL8 is a member of the CXC chemokine subfamily and plays a significant role in recruiting neutrophils towards the site of infection or damage. It seems that miR155 causes overexpression of IL-8 through inhibiting the expression of SHIP1,^33^ while miR-17 can reduce the expression of IL-8 in neutrophils through targeting it.^52^ Activation of inflammasome NLRP3 also causes overexpression of pro-inflammatory factors including IL-1β and IL-18. Possibly, miR-223 reduce the expression of IL-1β by targeting NLRP3.^53^ In addition, miR-223 also reduces the expression of IL-6 which is an important inflammatory cytokine. Further, this miRNA through targeting CCL3 and CXCL2 attenuate neutrophils chemotaxis.^54^ It seems that, miR-451 expression in neutrophils leads to diminished migration of neutrophils to the site of infection and inhibition of expression of the inflammatory factors such as, TNF-α, IL-1β, and cyclooxygenase-2.^55^

 The expression of miR-9 may increase in inflammatory gingival tissues. It seems that LPS through stimulating the MyD88-dependent signaling pathway causes activation of NF-κB and overexpression of miR-9 in neutrophils, where this miRNA controls inflammatory responses through inhibiting the activity of NF-κB.^56^

###  Macrophage, miRNAs and periodontal disease 

 After entering tissues, the monocytes present in the bloodstream convert to macrophages under the influence of special tissue growth factors, and these macrophages are very important in inflammatory diseases including periodontal disease. Macrophages, dendritic cells and monocytes belong to the mononuclear phagocyte system.^57^ Macrophage polarization can be pro-inflammatory or anti-inflammatory, whereby MIMΦ or M2MΦ phenotypes are formed. M1MΦ (classical activation state) is generally produced under inflammatory conditions and in response to a wide range of pathogens. However, M2MΦ (alternative activation state) is generally produced in the course of resolving inflammation and tissue regeneration, and has potent anti-inflammatory effects.^11,58^ GM-CSF causes induction of formation of MIMΦ or inflammatory macrophages. However, M-CSF causes stimulation of formation of M2MΦ or anti-inflammatory macrophages.^59^ miRNAs play a significant role in macrophage polarization. Overexpression of miR-155, miR-125b, and miR-21 supports the formation of MIMΦ. On the other hand, miR-146a causes enhanced formation of M2MΦ. Let-7c and miR-24 can cause conversion of M1MΦ to M2MΦ phenotype. However, miR-223 and miR-155 cause repolarization of M2MΦ to M1MΦ.^60^ miR-155 appear to be associated with LTB4 and macrophage activation. LTB4, an important inflammatory mediator and stimulator of macrophages, may be associated with the severity of periodontal disease. It seems that, LTB4 can up-regulate miR155 and this up-regulation may be involved in MyD88-dependent activation of macrophages.^61^

 It seems that, miR-125b can decrease the expression of TNF-α in different ways including attachment to TNF-α transcripts, inhibition of ERK1/2 activity, and intensifying deadenylation of TNF-α transcripts.^62^ Therefor investigation of the effects of this miRNA on macrophages in periodontal disease may be helpful.

 Porphyromonas gingivalis causes overexpression miR-132 in macrophages through activating the signaling pathway caused by TLR2/4 and NF-κB, where this miRNA exerts an excitatory effect on TNFα production.^63^ miR-21 can also be expressed in macrophages in response to Porphyromonas gingivalis LPS induced NF-κB activation. Through targeting PDCD4, miR-21 can attenuate NF-κB activity and thus repress the production of pro-inflammatory cytokines in macrophages. This miRNA also attenuates the polarization of macrophage to M2 phenotype by targeting STAT3.^64^ miR-147 and miR-24 are also other miRNAs which are associated with macrophages and periodontal disease. It seems that, up-regulation of miR-147 in periodontal tissue is associated with M1 macrophage polarization and this miRNA can enhance the expression of M1 macrophages markers including TNF-a, IL-12 and Nos2.^65^ miR-24 and miR-142-3p have an inhibitory effect on NF-κB activation and repress the production of pro-inflammatory cytokines in macrophages through targeting the components of the signaling pathways of PRRs.^66^

###  Dendritic cells, miRNAs and periodontal disease

 Dendritic cells are a member of mononuclear phagocyte system, which also lie in periodontal tissue. These cells have long fingerlike projections, which are similar to the dendrites of neurons, hence the name dendritic cells. These cells are the main cells that present antigen and capable to stimulating or inhibiting immune responses. miRNAs are in association with these cells, and expression of miRNAs in the cells may regulate the inflammatory responses in periodontal tissue. One of these miRNAs is miR-152. This miRNA is a negative regulator of antigen presentation by dendritic cells and can inhibit the production of TNF-α, IL-12, and IL-6. miR-148, similar to miR-152 can negatively regulate the innate immune response and is involved in the regulation of antigen presentation ability of dendritic cells.^67^ It seems that these miRNAs through targeting calcium/calmodulin dependent protein kinase II (CaMKII), which plays a significant role in dendritic cells maturity and functional, repress the production of TNF-α, IL-6, and IL-6 and enhance the expression of IFN-β.^68^ Therefore these miRNAs may be helpful targets for reducing the inflammation of dental pulps, and prevents development of periodontal disease. miR-146a and miR-155 are in relation with dendritic cells. Through attenuating the TLRs signaling pathway, miR-146a decrease the expression of cytokines.^69^ The expression of miR155 also increases throughout activation of dendritic cells. It seems that this miRNA inhibits the c-Fos expression in dendritic cells, whereby the maturity of dendritic cells and therefore antigen presentation by them are strengthen. On the other hand, c-Fos through inhibiting the activity of NF-κB causes inhibition of production of pro-inflammatory cytokines in dendritic cells. Therefore, miR-155 can also reinforce the production of pro-inflammatory cytokines in dendritic cells.^70^ miR-451 is another miRNA expressed in dendritic cells, which seems to reduce the expression of some cytokines including IL-6 and TNF-α as well as chemokines such as CCL5 and CCL3.^71^

 miR-142-3p is also expressed in dendritic cells and have inhibitory effects on production of inflammatory mediators in these cells. It seems that in response to LPS, miR-142-3p is expressed by dendritic cells and through targeting the IL-6 transcript, this miRNA can diminish the severity of inflammation.^72^

## MiRNAs, adaptive immunity and periodontal disease

 In addition to the innate immune response, the pathogens associated with periodontal disease can stimulate adaptive immune response. The adaptive immune responses are initiated upon detection of pathogens through antigen presenting cells such as dendritic cells.^73^ The adaptive immune system is composed of T and B lymphocytes. These lymphocytes are not able to directly confront antigens, and the cooperation between innate and adaptive immune systems is required to fight against pathogens.^74^ These lymphocytes respond to a wide range of microbial pathogens through expressing different antigen receptors. As stated earlier, B and T lymphocytes participate in the periodontitis-associated inflammatory processes.^75^ They are also highly interconnected with miRNAs. Indeed, MiRNAs in addition to regulating the innate immune responses are also involved in controlling the adaptive immune responses.

###  T lymphocyte, miRNAs and periodontal disease

 T lymphocytes or T cells originate from lymphoid progenitors and become mature in the thymus gland. Mature T cells are categorized into CD4 + and CD8 + T cells.^76^ Detection of antigen peptides presented by MHC class II and MHC class I by T-cell receptor (TCR) causes stimulation of differentiation of CD4 + T cell and CD8 + T cells, respectively.^77^

 Naïve CD4 + T cells can be differentiated into effector T cells and regulatory T cells (Tregs). Effector T cells include Th17, Th2, and Th1. The differentiation of these cells is determined by special cytokines, transcription factors, and co-stimulatory molecules. Naïve CD8 + T cells are differentiated into cytotoxic T cells.^78^ T lymphocytes are present in periodontal tissues. Since the bacteria which participate in periodontal disease are extracellular pathogens, CD4 + T cells play a significant role in immune response to these bacterial pathogens. Different MiRNAs are expressed in T cells which may be associated with periodontal disease. miR-146a is highly expressed in Th1 and Treg cells.^79^ This miRNA can act as an anti-apoptotic factor and by targeting Fas-associated death domain, it protects the T cells against activation induced cell death.^80^

 In addition, miR-146a by targeting protein kinases C epsilon (PRKCε) attenuate the differentiation of Th1-cells from human naïve CD4 + T-cells. PRKCε is part of the functional complex including PRKCε and signal transducer and activator of transcription 4 (STAT4), which is involved in the phosphorylation of STAT4 and thus differentiation of Th1-cells.^81^ The expression of miR-146a in Tregs cells plays a significant role in attenuation of Th1 response. STAT1 is one of the targets of miR-146a. STAT1 is necessary for differentiation of Th1 cells. It seems that, miR-146a may strengthens the regulatory function of Treg cells on Th1 response by targeting STAT1.^82^ miR-155 can be induced upon activation of naïve CD4 + T cells.^83^ miR-155 can promote the differentiation of Th1 cells, Th17 cells, and Treg cells by targeting various factors.^84^ Further, the expression of miR-155 is also induced during the activation of CD8 + T-cell, but its expression reduces quickly.^85^ SOCS1 inhibits the differentiation of CD4 + T cells to Th1, where enhanced expression of SOCS1 in T cells inhibits the signaling pathways of IFNγ and IL-12, thereby causing inhibited differentiation of Th1 cells and stimulated differentiation of Th2 cells.^86^ It seems that miR-155 through inhibiting the expression of SOCS1 stimulate the differentiation of Th1, Treg, and Th17 cells, and also strengthens the function of Th17 cells.^87^

 In addition to targeting SOCS1, miR-155 enhance the differentiation of Th17 through targeting ETS-1 transcription factor, and through attenuating the inhibitory effects of Jumonji AT Rich Interactive Domain 2 (Jarid2) causes improved function of Th17 cells and overexpression of cytokines by these cells.^88^ miR-17, miR-126 and miR-142-3p are among other miRNAs associated with Treg cells. It seems that miR-17 may attenuate the differentiation of iTreg cells, through targeting TGFβRII and cAMP-responsive element-binding protein1 (CREB1).^89^ miR-126 is highly expressed in Treg cells and its absence causes diminished inhibitory activity of these cells. It seems that absence of this miRNA stimulates the activity of PI3K/Akt pathway through enhancing the expression of p58B. Then, the stimulation of this pathway activity leads to altered inhibitory function of Treg cells through reducing the expression of Foxp3.^90^ It seems that Foxp3 can reduce the expression of miR-142-3p in Treg cells. This miRNA may attenuate the expansion of Treg cells following activation.^91^ Therefore, concerning the protective role of Treg cells in periodontal disease, as mentioned earlier, miR-142-3p may cause aggravation of the disease which needs to be investigated in future studies. miR-125b is also in association with T-cells. miR-125b can attenuate the differentiation of naïve CD4 + T cells to effector cells through targeting IL-2Rβ, IL-10Rα, IFN-γ, and PR domain zinc finger protein 1 (PRDM1), thereby this miRNA can act as anti-inflammatory agent.^92^ miR-125a is also highly expressed in Treg cells, and is required for enhancing the inhibitory function of these cells. Absence of miR-125a in Treg cells is associated with diminished expression of FOXP3 and overexpression of the molecules associated with Th1 cells such as IL-22, IFN-γ, IL-13, and Il-5. Further, miR-125a may cause reduction of IFN-γ, IL-13, and STAT3 by directly targeting them.^91^ miR-29 family which consists of miR-29c, miR-29a, and 29b is very important in the pathogenesis of periodontal disease. The expression of these miRNAs increases in gingival tissues of individuals with periodontitis.^61^

 The transcription factors Eomesodermin (EOMES) and T-bet which are involved in the production of IFN-γ and differentiation of Th1 cells are targets of miR-29, and miR-29 can repress the production of IFN-γ in Th1 cells by targeting these factors.^93,94^ In addition, miR-29 can directly target IFN-γ transcription in T cells and causing reduced expression of IFN-γ and, therefore, attenuated differentiation of Th1 cells.^95^ As stated earlier, IL-12 stimulate the production of IFN-γ in Th1 cells. Therefore, it has a significant role in the induction of polarization of Th1 cells. However, IL-4 causes stimulation of Th2 cells responses.^96^ In a study on rats, it was observed that miR-21 by inhibiting the production of IL-12 attenuate the differentiation of Th1 cells and stimulate the differentiation of Th2 cells.^97^ It seems that the expression of miR-21 also increases in human Treg cells, and act as a positive regulator of FOXP3 expression.^98^ Therefor this miRNA may has a protective role against periodontal disease progression, which need to be investigated in future studies.

 The effect of miR-181a on T cells is also very considerable. This miRNA by enhancing the activity of TCR signaling pathway molecules and inhibiting the negative regulators of this pathway such as DUSP6 phosphatase, may stimulate the proliferation and differentiation of T cells.^99^ In addition to T-bet, the Twist1 is also involved in both the differentiation and function of Th1 cells, and it seems that these transcription factors can stimulate the expression of miR-148a in Th1 cells. This miRNA through reducing the expression of Bim, a pro-apoptotic protein, leads to enhanced survival of Th1 cells and therefore their increased stability in chronic inflammation.^100^ It seems that miR-301a by inhibiting the expression of PIAS3, which is a STAT3 activity inhibitor, can activate STAT3, which resulted in the stimulation of Th17differentiation.^101,102^ Another microRNA that it expression is reduced in periodontal inflammation is miR-214.^103^ The expression of this miRNA increase, after activation of T cells. Through targeting phosphatase and tensin homolog (PTEN), this miRNA enhances the activity and proliferation of T cells.^104^

 Further, the overexpression of miR-214 through targeting mTOR, GBL, PDK1, and, AKT can attenuate the mTOR signaling pathway which resulted in stimulation of Treg cells differentiation. In addition, through targeting IL6R, AKT, STAT3, and STAT2, miR-214 causes inhibition of the JAK-STAT3 signaling pathway, thereby inhibiting the differentiation of Th17 cells.^105^ Generally, miRNAs influence different aspects of adaptive immunity ranging from differentiation of T cells to the function of these cells and secretion of cytokines. Concerning the role of T cells in periodontal disease, further focus on these miRNAs in studies associated with periodontal disease treatment can be effective.

###  B lymphocyte, humoral immunity, miRNAs and periodontal disease

 As with T lymphocytes, the initial progenitors of B lymphocytes originate from hematopoietic stem cells.^106^ However, unlike T cells, the maturation of B cells occurs in the bone marrow. Plasma cell is the final effector of B cells, which secrete antibodies, and require Th cells for activation.^107^ B cells can penetrate into periodontal tissues, and their elevated levels in these tissues are associated with advanced stages of periodontitis.^108^ Several miRNAs are also in relation to B cells and have significant importance in immunopathogenesis of periodontal disease. One of these miRNAs is miR-155. The expression of this miRNA increase following the activation of B cells in germinal centers. It seems that the absence of this miRNA in germinal centers leads to diminished number of B cells.^109^ Activation-induced cytidine deaminase (AID) is expressed in germinal center B cells and is required for immunoglobulin somatic hyper mutations and immunoglobulin class switch recombination. Immunoglobulin class switch recombination refers to a process through which B cells changes production of antibody from one type to another, such as from IgG to IgM. It seems that miR-155 reduces the expression of AID, thereby affecting the regulation of germinal-center reaction.^110,111^ Absence of miR-155 in B cells leads to diminished secretion of IgG1. Also, miR-155 by targeting Pu.1 causes inhibition of its negative effect in the production of IgG1-Switched Plasma cells.^112^ miR-181b also act as a negative regulator of AID expression in B cells, therefor the elevation of this miRNA levels can lead to impaired immunoglobulin class switch recombination.^113^ miR-150 is another miRNA which is in association with B cells and is highly expressed during maturation of B cells, but its expression is low in pro-B cells.^114^ It seems that the reduction of this miRNA through enhancing the expression of c-Myb, a transcription factor involved in the maturity of lymphocytes, leads to increased differentiation of B cells and enhanced humoral immune responses.^115^ Elevation of this miRNA in hematopoietic progenitors has been associated with impaired formation of mature B cells.^114^

 The transcription factors Blimp-1 and IRF4 are required for production of plasma cells. In addition, IRF4 reinforce the plasma cells survival. Presence of Blimp-1 is also essential for secreting antibodies by these cells.^116^ Through reducing the expression of Blimp-1 and IRF-4, miR-125b seems to cause inhibited differentiation of the B lymphocytes. However, miR-148a by overexpression of Blimp-1 and IRF4 stimulates the differentiation of plasma cells.^117^ All of these studies suggest the substantial role of miRNAs in the immune system. It seems that by targeting these small molecules, one can influence different aspects of immune response as well as immune-based diseases such as periodontal disease, and most probably in near future, further studies would be conducted on the role of these molecules in the pathogenesis of periodontal disease and other inflammatory disease, which will broaden our scope of current knowledge. Table 1 summarized the role of some important miRNAs in the pathogenesis of periodontal disease.

**Table 1:** Summary of miRNAs roles in the pathogenesis of periodontal disease

**MiRNAs**	**Targets**	**Effects**	**References**
miR-203	SOCS3	Porphyromonas gingivalis can increase the expression of miR-203 in HGECs and miR-203 can increase the production of cytokines and promote progression of periodontitis	^25^
miR-584	lactoferrin receptor	Attenuation of the anti-inflammatory effects of human lactoferrin in HGECs	^26^
miR-126	IL-8, CXCL1	Overexpression of IL-8 and CXCL1 in HGECs Enhancing the recruitment of immune cells	^28^
miR-155	SOCS1, MITF, SHIP1, c-Fos, Pu.1	Targeting SOCS1 and MITF, and attenuate osteoclast differentiation Enhancing differentiation of Th1, Treg, and Th17 cells by targeting SOCS1 Increasing IL 8 expression through inhibition of SHIP1 Enhancing maturity and antigen presentation of dendritic cells by inhibiting c-Fos expression Inhibiting the negative effect of Pu.1 on the production of IgG1-switched plasma cells	^33,36,70,87,112^
miR-146a	IRAK1, SMAD4	Repressing the secretion of pro-inflammatory cytokines in gingival fibroblasts, through reducing the expression of IRAK1, Attenuating TGF-β1/SMAD signaling and inhibiting fibroblasts differentiation	^41,45^
miR-126	TRAF6	Attenuating the NF-κB activity, repressing the secretion of pro-inflammatory cytokines in human gingival fibroblasts	^46^
miR-223	IL-1β and IL-6 CCL3 and CXCL2	Reducing the expression of IL-1β and IL-6 in gingival fibroblasts, Attenuating neutrophils chemotaxis, through targeting CCL3 and CXCL2, Alleviating inflammation	^47,54^
miR-181a	IL-8	Reducing the expression of IL-8 Attenuating progression of pulpitis to periodontitis	^50^
miR-17	IL-8	Reducing the expression of IL-8 in neutrophils	^52^
miR-9	NF-κB	Its expression is increased in inflammatory gingival tissue Controlling inflammatory responses through inhibiting the activity of NF-κB	^56^
miR-21	PDCD4,STAT3	Its expression is increased in response to *Porphyromonas gingivalis* LPS Attenuating NF-κB activity and repressing the production of pro-inflammatory cytokines in macrophages, through targeting PDCD4, -Attenuating the polarization of macrophage to M2 phenotype by targeting STAT3 Attenuating the differentiation of Th1cells and stimulating the differentiation of Th2 cells by inhibiting the production of IL-12	^64,97^
miR-147	TLRs signaling	Negative regulation of TLRs signaling pathway in the macrophages of periodontal tissue	^65^
miR-148/152	CaMKIIα	Attenuating antigen presentation of dendritic cells	^68^
miR-142-3p	Lipopolysaccharide	Lipopolysaccharide stimulation	^72^
miR-146a	PRKCε, STAT1	Attenuating the differentiation of Th1-cells by targeting PRKCε Enhancing regulatory function of Treg cells by targeting STAT1	^81,82^
miR-17	TGFβRII, CREB1	Attenuating the differentiation of Treg cells	^89^
miR-125b	IL-2Rβ, IL-10Rα, IFN-γ, PRDM1, Blimp-1, IRF4	Attenuating the differentiation of effector T cells, Inhibiting the differentiation of the B lymphocytes, through reducing the expression of Blimp-1 and IRF-4	^92,117^
miR-29	EOMES, T-bet, IFN-γ	The expression of this miRNAs appear to be increased in gingival tissues of patients with periodontitis miR-29 can repress the production of IFN-γ in Th1 cells by targeting EOMES and T-bet Attenuating the differentiation of Th1cells by direct targeting of IFN-γ	^61,93-95^
miR-148a	Bim, Blimp-1, IRF4	Enhancing the survival of Th1 cells, stimulating the differentiation of plasma cells by increasing the expression of Blimp-1 and IRF4	^100,117^
miR-301a	PIAS3	The expression of this miRNAs appear to be increased in inflamed gingival tissues Activating STAT3 and enhancing the differentiation of Th17 cells	^101,102^
miR-214	IL6R, AKT STAT3 STAT2	Inhibiting JAK-STAT3 signaling Attenuating the differentiation of Th17cells	^105^
miR-181b	AID	Negative regulation of AID in B cells Impairing immunoglobulin class switch recombination	^113^

## Conclusion and future directions

 Periodontal disease is an inflammatory disease in which the reaction of the immune system to the bacteria accumulated in the gum line leads to inflammation of the gum and in advanced stages causes loosening and loss of teeth. miRNAs play a significant role in the pathogenesis of periodontal disease. These molecules influence different aspects of the immune response, and are in association with the immune system as well as the periodontium cells including GECs and periodontal ligament cells. Some of these miRNAs can be potential therapeutic targets for treating the periodontal disease. For example, one can consider usage of RNA mimics and strengthening the function of miRNAs which are involved in improving the inhibitory function of Treg cells, reducing inflammatory response, reducing the expression of TNFα, and compromising osteoclastogenesis as a potential therapeutic approach for offsetting the course of progression of periodontal disease. Although so far the efficacy of RNA mimics and antagomirs has not been evaluated as a new therapeutic approach in treating periodontal disease, in some other inflammatory diseases, positive therapeutic effects have been observed. Concerning the substantial role of miRNAs in the immunopathogenesis of periodontal disease which was discussed in this review paper in detail, it seems that usage of RNA mimics and antagomirs can be noted as a new therapeutic approach for effective treatment of periodontal disease. Finally, concerning the very high prevalence of this disease, more extensive studies should be conducted.

## Acknowledgments

 The Authors would like to thank Clinical Research Development Unit, Shohada Hospital, Tabliz University of Medical Sciences for their kind support.

## Competing Interests

 Authors declared no conflict of interests.

## Ethical Approval

 This article does not contain any studies with human participants or animals performed by any of the authors.
